# Comparison of Morphological Characteristics of Antennae and Antennal Sensilla among Four Species of Bumblebees (Hymenoptera: Apidae)

**DOI:** 10.3390/insects14030232

**Published:** 2023-02-26

**Authors:** Chang-Shi Ren, Zhi-Min Chang, Zhi-Yun Zu, Lei Han, Xiang-Sheng Chen, Jian-Kun Long

**Affiliations:** 1 Key Laboratory of Animal Genetics, Breeding and Reproduction in the Plateau Mountainous Region, Ministry of Education/Guizhou Provincial Key Laboratory of Animal Genetics, Breeding and Reproduction/College of Animal Science, Guizhou University, Guiyang 550025, China; 2 Institute of Entomology/Special Key Laboratory for Developing and Utilizing of Insect Resources, Guizhou University, Guiyang 550025, China

**Keywords:** *Bombus*, antennae, antennal sensilla, SEM, three castes, morphology, distribution

## Abstract

**Simple Summary:**

Bumblebees, as important pollinating insects, play a vital role in maintaining natural and agricultural ecosystems. Its antennae with sensilla can guide in selecting a nectariferous source, locating nest sites, and communicating between individuals. To explore how bumblebees detect and receive chemical signals from nectariferous plant and foraging behavior, the morphology of antennae with sensilla, including antennal length and type, distribution, and number of antennal sensilla were compared in four bumblebee species, *Bombus atiapes*, *Bombus breviceps*, *Bombus flavescens*, and *Bombus terrestris* through scanning electron microscopy. The characteristics and differences in the total antennal length, and length of scape, pedicel, and flagellum were recorded among three castes and four species. Furthermore, 13 major types of antennal sensilla in total were observed, including trichodea sensilla (TS A-E), placodea sensilla (PS A-B), basiconica sensilla (BaS), coeloconica sensilla (COS A-B), chaetic sensilla (CS A-B), and Böhm sensilla (BS), of which chaetic sensilla B (CS B), only observed in females of *B. atripes*, was firstly reported in Apidae. The characteristics and differences in the distributions and number of sensilla were also found among three castes and four species. Therefore, this study may help to understand the effects of antennae with sensilla in the coevolution of species and the external environment.

**Abstract:**

Bumblebees, as pollinators, play an important role in maintaining natural and agricultural ecosystems. Antennae with sensilla of bumblebees as social insects have essential effects in foraging, nest searching, courting, and mating, and are different in species and sexes. Previous studies on the morphology of antennae and sensilla in bumblebees have been limited to a few species and a single caste. To better understand how bumblebees detect and receive the chemical signal from nectariferous plants and foraging behavior, the morphology of antennae with sensilla, including the antennal length, and type, distribution, and number of antennal sensilla in four species, *Bombus atripes*, *Bombus breviceps*, *Bombus flavescens*, and *Bombus terrestris* was compared by scanning electron microscopy (SEM) herein. The total antennal length of queens are the longest and workers are the shortest in three castes, and in four species the longest of the total antennal length among three castes all are in *B. flavescens*, which is significantly longer than other species (*p* < 0.05) and the length of the scape in queens and workers are both longer than males, significantly different in queens (*p* < 0.05), and not significantly different in workers (*p >* 0.05), and the length of flagellums in females are not always shorter than males, of which the length of flagellms in queens of *B. flavescens* are significantly longer than males (*p* < 0.05), and the length of pedicel and all flagellomeres varies among species and castes. A total of 13 major types of sensilla in total were observed, including trichodea sensilla (TS A-E), placodea sensilla (PS A-B), basiconica sensilla (BaS), coeloconica sensilla (COS A-B), chaetic sensilla (CS A-B), and Böhm sensilla (BS), of which chaetic sensilla B (CS B), only observed in females of *B. atripes*, was firstly reported in Apidae. Moreover, the number of all sensilla was the most in males, the least was in workers, the number of sensilla varies within castes and species. Furthermore, the morphological characteristics of antennae and the potential functions of sensilla are discussed.

## 1. Introduction

The antennae of insects are highly intricate sensory structures involved in the primary sensory organs receiving various environmental signals, such as mechanical sensilla, chemical sensilla, thermo- and hygroreceptive sensilla [1,2]. They play an important role in insect life behaviors, such as habitat selection, food searching, mating, oviposition sites, and intraspecific and interspecific recognition [3,4,5,6]. Previous studies have shown that the type and distribution of sensilla in antennae are related to sex [7,8,9], feeding [10,11], and whether they sojourned [12,13], which are decided at the stage of insect morphogenesis [14,15]. Even the types, numbers, and distribution of antennal sensilla are diverse in different castes in the same species [16,17]. Identifying and characterizing antennae and sensilla in insects contributed to clarifying the value of antennal characters in biological evolution and the olfactory recognition mechanisms of behavioral control [18].

Bumblebees (*Bombus* spp.) (Hymenoptera: Apidae) are important pollinators of many wild flowering plants and crops, and play a significant role in maintaining natural and agricultural ecosystems [19,20]. There are about 250 species distributed worldwide, especially in the high mountains of the Northern Hemisphere [21], with approximately 125 species in China [22,23]. As social insects, the antennae with sensilla of bumblebees have an essential role in foraging, nest searching, courting, and mating, and especially in locating nectariferous plants and identifying companions and invaders [24]. For instance, *Bombus terrestris* can decide whether to visit a flower by sensing its electric charge through antennae [25]. Therefore, the antennae with sensilla of bumblebees are closely related to its behavior.

Previous studies have reported the morphology of antenna with sensilla in bumblebees referring to the *Bombus morrisoni* female (subgenera: *Cullumanobombus*) with eight types of sensilla, including sensillum trichodeum A, B, C/D, sensillum ampullaceum, sensillum coeloconicum, sensillum basiconicum, sensillum placodeum, and seta [13]; and 12 species of nine subgenera including *Alpinobombus*, *Bombias*, *Bombus*, *Crotchiibombus*, *Cullumanobombus*, *Fraternobombus*, *Melanobombus*, *Pyrobombus*, and *Separatobombus*, with sensilla campaniform recorded firstly in bumblebees [16], and eight species belonging to four subgenera with Böhm sensilla as a new type and sensilla basiconica A recorded firstly in [26], and *Bombus hypocrite* with bud-like sensilla and finger-like sensilla firstly reported in [27]. In addition, the types and distributions of antennal sensilla in *Bombus pauloensis* were compared in three castes, with females having higher diversity of types than males [8]. Compared with other species of bumblebees, the native three species *Bombus atripes*, *Bombus breviceps*, and *Bombus flavescens*, belonging to the subgenus *Thoracobombus*, *Alpigenobombus*, and *Pyrobombus*, respectively, are the most abundant and essential indicator species in southern China [28,29]. These three Asian bumblebee species are vital pollinators for many plants and play critical roles in local ecosystems. For instance, *Bombus atripes* is the primary pollinator for *Aralia chinensis*, *Verbena officinalis*, and *Salvia leucantha* [30,31]; *Bombus breviceps* is the primary pollinator for *Amomum subulatum*, *Buddleja officicinalis*, and *Hypericum perforatum* [30,32]; and *Bombus flavescens* is the primary pollinator for *Arctium lappa*, *Helianthus annuus*, and *Alcea rosea* [30]. In addition, the commercial *Bombus terrestris* belonging to the subgenus *Subterraneobombus* [33] has been deliberately introduced into many countries including China for crop pollination [34]. The alien species *B. terrestris* has led to biological invasion in many countries and may be impacting indigenous bumblebees and ecosystems in southern China [35,36,37]. Previous studies on bumblebee antennae with sensilla have only demonstrated several species and only male castes [16], and have not included the above three native species and females of *B. terrestris*, which may be unfavorable to the understanding and identification of bumblebees and recognition mechanisms of behavioral control.

Therefore, in this paper the antennae of four species, *B. atripes*, *B. breviceps*, *B. flavescens*, and *B. terrestris*, were observed through scanning electron microscope (SEM) to clarify the differences in the morphology, the types, and distribution of antennae with sensilla among three castes and the above four species.

## 2. Materials and Methods

### 2.1. Insect Rearing and Sample Collection

The queens’ source of the four species used in this study are in Table 1. The queens and colonies were reared in the laboratory of the College of Animal Science, Guizhou University, Guiyang, China. Feeding methods were based on the study by Ryder [38]. The samples and materials examined, including the queens, workers, and drones, were collected and deposited at the Institute of Entomology, Guizhou University, Guiyang, China (IEGU). The bumblebee species were identified by the references [21,31] according to the external morphology and male genitalia. 

### 2.2. Scanning Electron Microscopy

Five samples for each caste were utilized for SEM observations. First, antennae were dissected from the head under Nikon SMZ745, and then cleaned using an ultrasonic cleaner (AK-031SD, Yuclean, Shenzhen, China) for 15 min to wash away the remaining pollens. Then the samples were transferred to 4% glutaraldehyde and fixed for 24 h. Next, the samples were dehydrated with 75%, 80%, 90%, and 95% ethanol for one hour, respectively, then in 100% ethanol solutions dehydrated for ten days and were dried in a 40 °C dryer for 10 h. Dried antennae were adhered to the microscope stub by conductive silver glue and gold-sputtered for 3 min in a smart coater; the prepared samples were scanned and taken photos using a scanning electron microscope (JCM 6000, JEOL, Tokyo, Japan) with the acceleration voltage set as 5–15 kV. 

### 2.3. Data Processing and Statistical Analysis

The morphology, number, distribution, and length and diameter of antennae and sensilla in the four bumblebee species were obtained from SEM images. The antenna length is measured by SEM built-in software and the number of sensilla is calculated using Adobe Photoshop CS 2018, referring to [39]. Data analysis was completed using analysis of variance (ANOVA) and Student’s *t*-test by SPSS V. 26.0 software (IBM, Portsmouth, UK). Tukey post-hoc tests were used to confirm where significant differences occurred. The results are expressed as the mean ± SE. Values of *p* less than 0.05 and 0.01 indicate a significant difference and an extremely significant difference, respectively. The morphological terminology and classification of antennal sensilla follow Schneider [15]. The graphs were fitted by GraphPad Prism V 9.4.1 software (GraphPad Software, San Diego, CA, USA). 

## 3. Results

### 3.1. Overall Morphology of Antennae

**Figure 1:**
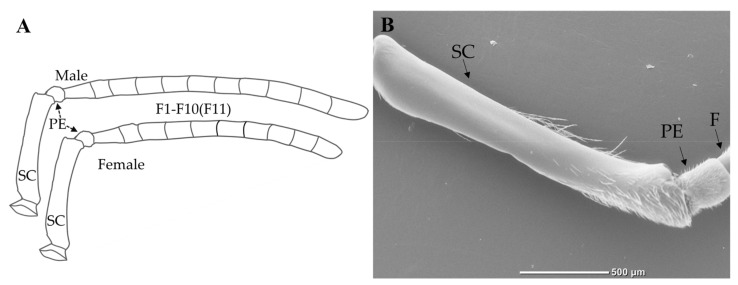
Antennal morphology of bumblebees. (**A**) An overview in male and female; (**B**) ventral view of a scape. SC: scape; PE: pedicel; F: flagellum; F1–F10(F11): flagellomere 1–10 or flagellomere 11.

**Figure 2:**
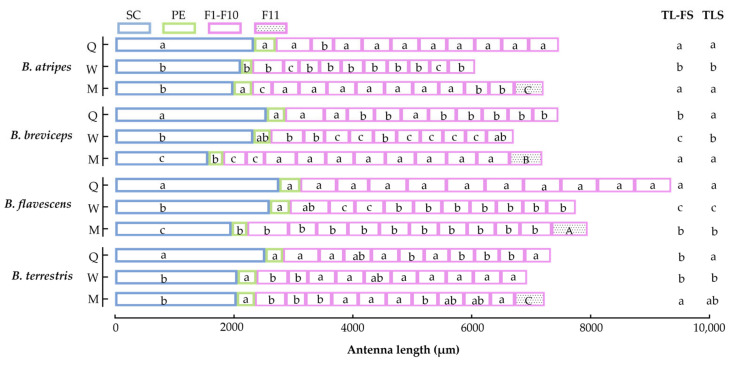
The mean length of the antennae in three castes of four species of bumblebees. SC: scape; PE: pedicel; F1–F10: flagellomere 1–10; F11: flagellomere 11; TL-FS: total length of the flagellum in same species; TLS: total length of the antenna in same species. The lowercase letters a, b, and c indicate the differences in three castes of the same species; The capital letters A, B, and C indicate the differences between F11 in the four species. Bars with the same letter are not significantly different (*p >* 0.05).

**Figure 3:**
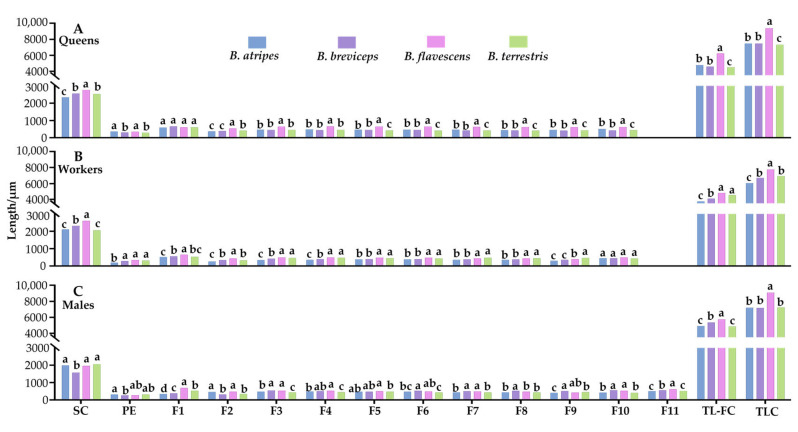
The mean length of antennomeres in four species of three castes of bumblebees. (**A**) The length of antennae in queen of four bumblebee species; (**B**) The length of antennae in worker of four bumblebee species; (**C**) The length of antennae in male of four bumblebee species. SC: scape; PE: pedicle; F1–F11: flagellum 1–11; TL-FC: total length of the flagellum in same caste; TLC: total length of the antenna in the same caste. Bars with the same letter are not significantly different (*p >* 0.05).

The knee-shaped antennae of the *B. atripes*, *B. breviceps*, *B. flavescens*, and *B. terrestris* are similar in three castes, all consisting of a scape (SC), a pedicel (PE), and a flagellum (F), including ten segments in females and eleven in males (drone), named as F1–F10 and F11, respectively (Figure 1A,B). The total antennal length of the four species is 6053.70 ± 61.20 μm to 9358.60 ± 112.93 μm; the queen of *B. flavescens* has the longest, and the workers of *B. atripes* has the shortest (Figure 2 and Figure 3). In three castes of all species, the mean total antennal length in queens is longer than drones and workers, the queen is significantly different from the workers (*p* < 0.05), and the workers have the shortest antennae (Figure 2). In the same species, there is no significant difference (*p >* 0.05) in queens and drones (except in *B. flavescens*), while a significant difference in queen and worker, and drone and worker (except in *B. terrestris*) was found in the four species (*p* < 0.05). In the same caste, the total antennae length ranges from 7334.40 ± 59.44 μm to 9358.60 ± 112.93 μm in the queen, with *B. flavescens* having significantly longer antennae than other species (*p* < 0.05), and *B. terrestris* significantly shorter than other species (Figure 3A). Ranging from 6053.70 + 61.20 μm to 7752.20 + 96.17 μm in workers, and the longest and shortest antennae are found in *B. flavescens* and *B. atripes*, respectively. Among workers, the length of antennae in *B. flavescens* is significantly longer (*p* < 0.05) than other species, and *B. atripes* is significantly shorter (*p* < 0.05) than other species (Figure 2). In drones, the length ranges from 7187.50 ± 62.76 μm to 9074.00 ± 131.26 μm, the longest in *B. flavescens*, which is significantly longer (*p* < 0.05) than other species (Figure 3C).

#### 3.1.1. Scape (SC)

The SC is slightly arched to the inner side, with the length approximately equaling 1/3–1/2 of the whole antenna (Figure 1A), with all sensilla including chaetic sensilla and branched setae distributed in the dorsal, inner, and outer sides, and smooth in the ventral side, only the chaetic sensilla are distributed near the apical part (Figure 1B). The mean length of SC in the four species ranges from 1566.60 ± 31.65 μm to 2760.60 ± 34.52 μm, the longest is in the queen of *B. flavescens*, and shortest in the males of *B. breviceps* (Figure 2). In the same species, the longest SC was found in the queen, the shortest in the male, and the SC is significantly longer in the queen than the males (*p* < 0.05; Figure 2), and only the workers have significantly (*p* < 0.05) longer SC than the males in *B. breviceps* and *B. flavescens*. In the same caste, the total antennae length ranges from 2333.80 ± 18.96 μm to 2760.60 ± 34.52 μm in queens; *B. flavescens* has significantly longer SC than other species, *B. atripes* has significantly shorter SC than other species (*p* < 0.05; Figure 3A). In workers the scape ranges from 2062.20 ± 10.06 μm to 2602.60 ± 25.34 μm; the longest and shortest are in *B. flavescens* and *B. terrestris*, respectively, and the length of SC in *B. flavescens* is significantly longer than other species, and the scape in *B. breviceps* is significantly longer (*p* < 0.05) than *B. atripes* and *B. terrestris* (Figure 3B). In drones ranging from 1566.60 ± 31.65 μm to 2047.60 ± 25.28 μm in scape length, the shortest scape is in *B. breviceps*, which is significantly shorter than other species (*p* < 0.05; Figure 3C).

#### 3.1.2. Pedicel (PE)

The PE is extremely short and slightly rounded, with all sensilla including trichodea sensilla, chaetic sensilla, and Böhm sensilla distributed near the base and middle, and smooth in the basal and apical margins. The length of the pedicel ranges from 191.00 ± 18.00 μm to 367.00 ± 7.19 μm in the four species. The longest and shortest length of PE is in the queens and males of *B. atripes*, respectively. The same species has no evident different trend (Figure 2). In the same caste, the length of PE ranges from 299.00 ± 9.27 μm to 367.00 ± 7.19 μm in queen, the longest and shortest are found in *B. atripes* and *B. terrestris*, respectively. *B. atripes* and *B. flavescens* have significantly longer PE than *B. breviceps* and *B. terrestris* in queens (*p* < 0.05; Figure 3A). In workers, the length of PE ranges from 191.0 ± 18.00 μm to 339.20 ± 4.85 μm, the longest and shortest are found in *B. flavescens* and *B. atripes*, respectively, and *B. atripes* has a significantly shorter (*p* < 0.05) PE than other species (Figure 3B). In males, ranging from 251.10 ± 12.32 μm to 315.70 ± 9.11 μm, *B. atripes* (longest) has a significantly longer (*p* < 0.05) PE than *B. breviceps* (shortest) (Figure 3C).

#### 3.1.3. Flagellum (F)

The flagellum is a slightly flattened cylinder (Figure 1A). The mean length of flagellums ranges from 3746.30 ± 66.06 μm to 6242.10 ± 109.62 μm in the four species. The longest of flagellum is in the queen of *B. flavescens*, and the shortest is in the workers of *B. atripes*. In the same species, the longest flagellum length was found in the males (except in *B. flavescens*, where the longest is in the queen) and the shortest in the workers among the four species. There is a significant difference (*p* < 0.05) between males and workers in all species. In addition, the length of the flagellum in queens and workers has a significant difference (*p* < 0.05), except in *B. terrestris*; queens and males differ significantly (*p* < 0.05), except in *B. atripes* (Figure 2). In the same caste, the total flagellum length ranges from 4502.60 ± 40.63 μm to 6242.10 ± 109.62 μm in queens, and *B. flavescens* is significantly longer than other species, *B. terrestris* flagellums are significantly shorter than other species (*p* < 0.05; Figure 3A). Workers’ flagellums range from 3746.30 ± 66.06 μm to 4810.40 ± 70.12 μm, the longest and shortest in *B. flavescens* and *B. atripes*, respectively. In workers, the flagellum length in *B. atripes* and *B. breviceps* is significantly shorter than other species (*p* < 0.05; Figure 3B). In drones, ranging from 4875.60 ± 49.62 μm to 5727.60 ± 74.70 μm, the longest flagellum is in *B. flavescens*, which is significantly longer than other species, the flagellum length of *B. breviceps* is significantly longer than *B. atripes* and *B. terrestris* (*p* < 0.05; Figure 3C). Each segment of flagellum was compared in three castes of the four species. In the same species, F1 in the female is longer than male, and only partial species with significant differences, and the length of other flagellomeres varies among species and castes (*p* < 0.05; Figure 2). In the same caste, the length of all flagellomeres in the queen is longest in *B. flavescens*, which are significantly longer than other species (except in F1) (*p* < 0.05; Figure 3A). In workers, the length from F1 to F7 and F10 in *B. flavescens* is also the longest, while the length from F8 to F9 in *B. terrestris* is the longest (Figure 2 and Figure 3B). In males, F1 to F5 in *B. flavescens* is the longest, while F6 to F10 in *B. breviceps* (Figure 2 and Figure 3C). In F11, the length of *B. atripes* and *B. terrestris* is significantly shorter (*p* < 0.05) than *B. flavescens* and *B. breviceps* (Figure 2 and Figure 3C). In the same species, the queen’s F1 (except in *B. flavescens*) is the longest and shortest in F2. In workers, the longest is in F1 and the shortest in F2 (except for *B. flavescens*). In males, the longest is F11 in *B. atripes* and *B. breviceps*, F1 in *B. flavescens* and *B. terrestris*, and the shortest is F2 in *B. breviceps* and *B. terrestris*, F1 in *B. flavescens*, and F9 in *B. terrestris* (Table 2).

### 3.2. General Description of Morphology, Distribution and Number of Antennal Sensilla 

**Figure 4:**
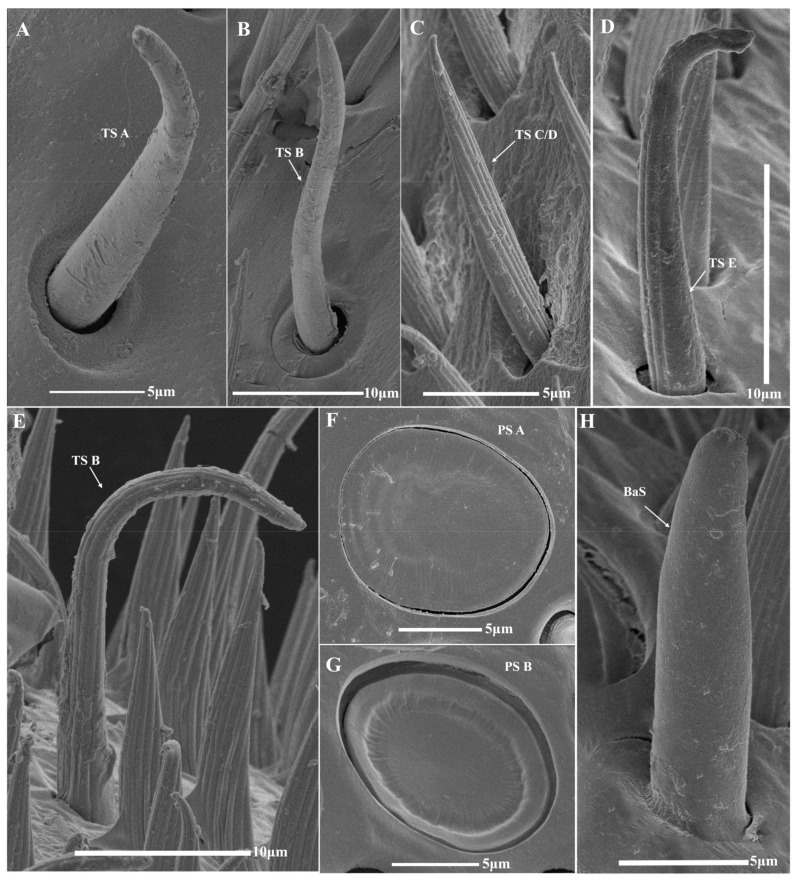
SEM images of antennal sensilla in bumblebees. (**A**) F2 of *B. breviceps*; (**B**) F6 of *B. flavescens*; (**C**) F2 of *B. atripes*; (**D**,**E**) F10 of *B. terrestris*; (**F**–**H**) F6 of *B. breviceps*. TS A: trichodea sensilla A; TS B: trichodea sensilla B; TS C/D: trichodea sensilla C or D; TS E: trichodea sensilla E; PS A: placodea sensilla A; PS B: placodea sensilla B; BaS: basiconica sensilla.

**Figure 5:**
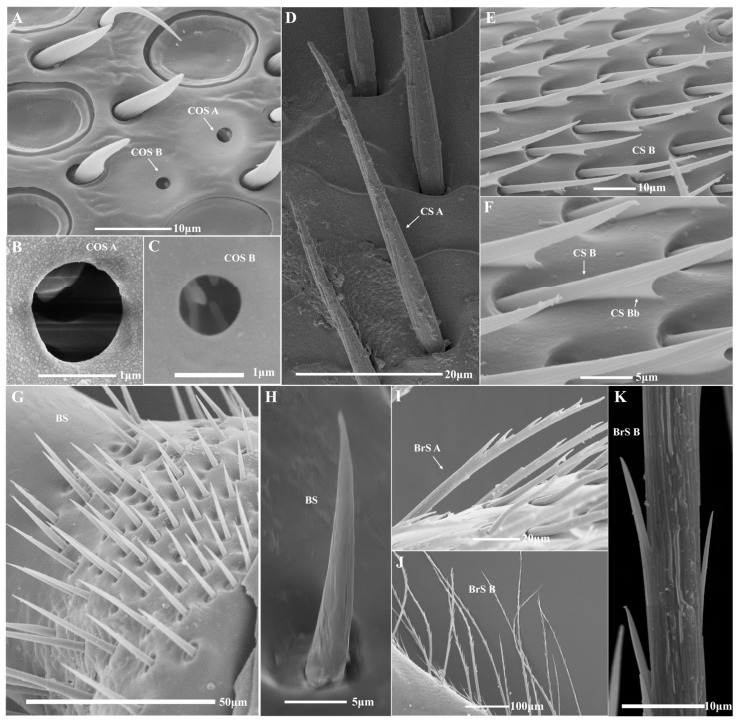
SEM images of antennal sensilla in bumblebees. (**A**–**C**) F9 of *B. flavescens*; (**D**) SC of *B. atripes*; (**E**,**F**) F1 of *B. atripes*; (**G**,**H**) Junction of SC and PE of *B. flavescens*; (**I**–**K**) SC of *B. terrestris*. COS A: coeloconicum sensilla A; COS B: coeloconicum sensilla B; CS A: chaetic sensilla A; CS B: chaetic sensilla B; CS Bb: extended basal structure of CS B; BS: Böhm sensilla; BrS A: branched sensilla A; BrS B: branched sensilla B.

**Figure 6:**
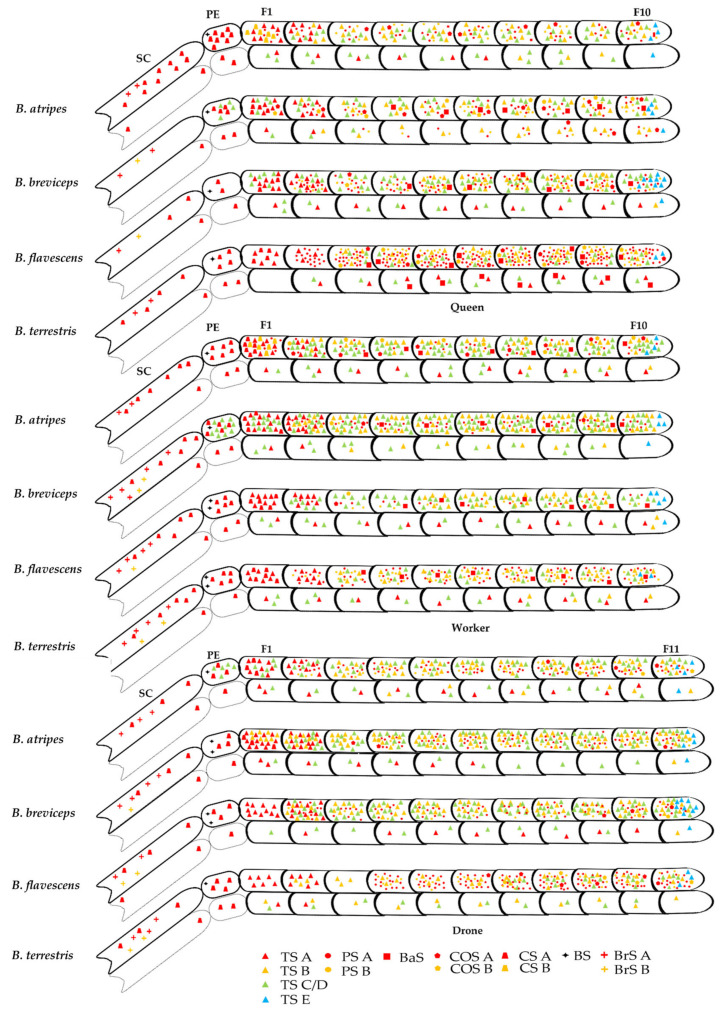
Distribution of antennal sensilla in four species of bumblebees. The solid and dotted lines represent the dorsal side and ventral side, respectively.

Trichodea sensilla (TS A–E), placodea sensilla (PS A, B), basiconica sensilla (BaS), coeloconica sensilla (COS A, B), chaetic sensilla (CS A, B), Böhm sensilla (BS), and branched setae (BrS A, B) were identified in four species of bumblebees, of which chaetic sensilla B (CS B) was firstly recorded in bumblebees (Figure 4 and Figure 5). There are many similarities in sensilla types and distribution among the three castes of all species, and differences between the sexes (Figure 6). For each segment, similarities exist in the scape (SC) with sensilla CS A and BrS A, B, and the PE with CS A and BS, and the flagellum with other sensilla on the dorsal (Table 3). For sensilla types, the similarities in trichodea sensilla A (TS A) are restricted in F1 and F2, trichodea sensilla E (TS E) is restricted in the apical segment (F10 in females and F11 in males), chaetic sensilla A (CS A) in SC, PE, and F1, Böhm sensilla (BS) only in PE, and BrS A, B is distributed in SC (Table 3). In the same species, the distribution of the sensilla type showed high similarity between the queen and worker, which differs from the drone in TS-C/D, TS E, PS A, B, CS B, COS A, B, and BaS, especially males without BaS (Table 3). In three castes of all species, the distribution of sensilla type and setae in queens and workers showed high similarity in TS A, TS E, CS A, BS, and BrS A among the four species (Table 3); and differences in TS B-C/D, PS A, B, BaS, COS A, B, CS B, and BrS B among partial species or all species, especially *B. atripes* without BrS B. The distribution of sensilla type and setae in drone showed high similarity in TS A, TS E, BaS, CS A, B, BS, and BrS A among the four species. There are many similarities in sensilla types and their distribution among these three castes of all species, and differences between the sexes. The TS and PS were generally richer among all sensilla in the four species, while BS and BrS were less rich. There are obvious differences in the numbers of sensilla among the three castes of the four species. The most and least number of sensilla are placodea sensilla A (PS A) of *B. terrestris* and branched setae B (BrS B) of *B. breviceps* in the queen. In the same species, the greatest number of all sensilla exists in the male, and the least in the worker. In the same caste, the number of sensilla varies in castes and species (Table 3). 

#### 3.2.1. Trichodea Sensilla (TS)

TS are hairy, and taper to the tip. Five types of TS (TS A–E) were observed according to their morphological features.

TS A are blunt-tipped hairs on the basal fossa, the apical part is slightly curved and tapered distally with a smooth surface and without a hole on the surface and top (Figure 4A). The sensilla are distributed in the inner and dorsal sides from the base to the end of F1 and F2 (Figure 6, Table 3). In the same species, the greatest number of TS A is in the males, more than the workers and queens. In the same caste, the number of TS in *B. flavescens* is significantly more than in *B. breviceps* and *B. terrestris* in the queen (*F* = 42.607, df = 3, *p* < 0.05; Table 3), *B. breviceps* has significantly more than other species in workers (*F* = 33.604, df = 3, *p* < 0.05; Table 3), and *B. breviceps* and *B. flavescens* have significantly more than *B. atripes* and *B terrestris* in males. The length of TS A is from 22.47 ± 1.36 μm to 31.57 ± 1.16 μm in the queen, from 17.24 ± 1.28 μm to 29.87 ± 1.67 μm in workers, and from 20.04 ± 1.02 μm to 23.82 ± 1.31 μm in males (Table 4). The width of the basal diameter is from 3.24 ± 0.21 μm to 4.84 ± 0.38 μm in the queen, from 2.72 ± 0.20 μm to 4.12 ± 0.28 μm in workers, and from 2.89 ± 0.08 μm to 4.63 ± 0.21 μm in males (Table 4).

TS B are blunt-tipped hairs on the basal fossa, gradually thin, the middle part is approximately S-bend curved, and they have shallow longitudinal grooves in the surface, and no hole on the surface and top (Figure 4B). TS B is overall finer than TS A and distributed on all sides of flagellum F2–F10 or partial flagellomeres (Figure 6, Table 3). In the same species, the males have the most TS B in three species (except in male workers of *B. breviceps*). In the same caste, the number of TS B in *B. flavescens* is significantly more than in *B. breviceps* and *B. terrestris* in the queen (*F* = 16.755, df = 3, *p* < 0.05, Table 3), *B. breviceps* have significantly more than other species in workers (*F* = 141.487, df = 3, *p* < 0.05, Table 3), and *B. terrestris* have significantly less than other species in males (*F* = 32.660, df = 3, *p* < 0.05, Table 3). The length of sensilla TS B is from 11.06 ± 1.04 μm to 19.89 ± 0.81 μm in queens, from 13.30 ± 0.37 μm to 25.80 ± 1.87 μm in workers, and from 11.04 ± 0.48 μm to 15.46 ± 0.63 μm in males (Table 4). The width of basal diameter is from 1.41 ± 0.07 μm to 3.12 ± 0.17 μm in queens, from 1.45 ± 0.09 μm to 2.17 ± 0.10 μm in workers, and from 1.71 ± 0.11 μm to 1.96 ± 0.09 μm in males (Table 4).

TS C/D is like TS B, but the apical part is straight and with deep longitudinal grooves, without a hole on the surface and top, and no basal fossa at the base (Figure 4C). Overall, it is thinner than TS A but thicker than TS B. The sensilla are mainly distributed on the dorsal side in all flagellomeres or partial flagellomeres, and rarely extends to PE (Figure 6, Table 3). In the same species, TS C/D is the most numerous in male, more than the workers and queens. In the same caste, the number of TS C/D in *B. flavescens* is significantly more than other species in queens and males, and *B. breviceps* has significantly more than other species in workers (*F* = 49.175, df = 3, *p* < 0.05; Table 3). The length of sensilla TS C/D is from 21.71 ± 1.36 μm to 34.03 ± 2.09 μm in queens, from 17.79 ± 0.73 μm to 32.25 ± 1.47 μm in workers, and from 18.63 ± 0.33 μm to 22.81 ± 0.76 μm in males (Table 4). The width of basal diameter is from 2.31 ± 0.15 μm to 4.29 ± 0.23 μm in queens, from 2.26 ± 0.19 μm to 3.47 ± 0.11 μm in workers, and from 2.21 ± 0.06 μm to 2.85 ± 0.22 μm in males (Table 4).

TS E is similar to TS A, but the apical part is sharply curved at the base, its size is close to TSB, with deep longitudinal grooves, without a hole on the surface and top, and the basal fossa is absent at the base (Figure 4D,E). It is only distributed on all sides of the terminal segment of the flagellum, F10 in females and F11 in males (Figure 6, Table 3). In the same species, the greatest number of TS is in males, more than workers and queens. In the same caste, the number of TS in *B. flavescens* is significantly more than other species in queens and males; in workers, the number of TS E in *B. flavescens* is significantly more than *B. atripes* and *B. terrestris* (*F* = 14.648, df = 3, *p* < 0.05; Table 3). The width of basal diameter is from 1.44 ± 0.09 μm to 1.84 ± 0.09 μm in queens, from 1.29 ± 0.07 μm to 1.93 ± 0.10 μm in workers, and from 1.12 ± 0.08 μm to 1.82 ± 0.07 μm in males (Table 4).

#### 3.2.2. Placodea Sensilla (PS)

PS is an oval or circular plate with a wide border (Figure 4F,G). Two types of placodea sensillum, PS A and PS B, were observed according to their morphological features. In females, PS is distributed in all sides of the antenna, while in males PS is not distributed in the ventral sides (Figure 6). 

PS A is an oval to nearly circular disc, which has a narrow border with some weak striae extending to the center of the shallower disc, the longest dimension is parallel to the long axis of the antennae (Figure 4F). PS A is distributed in F2–F10 in females (extending to F1 in *B. atripes*) and F4–F11 in males (extending to F2 and F3 in *B. atripes* and *B. flavescens*) (Figure 6, Table 3). In the same species, the greatest number of PS A is in the queen of *B. flavescens* and *B. terrestris*, while the most is in males of *B. atripes* and *B. breviceps*. In the same caste, the number of PS A in *B. terrestris* is significantly more than other species in all three castes (queen: *F* = 525.978, worker: *F* = 133.203, male: *F* = 28.986, df = 3, *p* < 0.05, Table 3). The length of sensilla PS A is from 13.96 ± 0.24 μm to 14.86 ± 0.24 μm in queens, from 12.74 ± 0.34 μm to 15.82 ± 0.33 μm in workers, and from 12.77 ± 0.34 μm to 13.40 ± 0.24 μm in males (Table 4). The width of basal diameter is from 9.83 ± 0.23 μm to 10.38 ± 0.35 μm in queens, from 8.52 ± 0.16 μm to 9.42 ± 0.34 μm in workers, and from 7.88 ± 0.35 μm to 9.61 ± 0.37 μm in males (Table 4).

PS B is similar to PS A, but with a wide border with some weak striae extending to the center of the deeper disc (Figure 4G). The distribution of PS B is like PS A, and PS A, the only difference is in the female of *B. atripes* and *B. breviceps* (Figure 6, Table 3). In the same species, the greatest number of PS B is in queens, more than males and workers (Table 3). In the same caste, the number of PS B in *B. terrestris* is significantly more than other species in queens and males; in workers, the number of PS B *B. flavescens* is significantly more than other species (*F* = 46.302, df = 3, *p* < 0.05, Table 3). The length of sensilla PS A is from 11.36 ± 0.21 μm to 14.19 ± 0.48 μm in queens, from 12.40 ± 0.40 μm to 14.47 ± 0.18 μm in workers, and from 8.87 ± 0.49 μm to 13.57 ± 0.57 μm in males (Table 4). The width of basal diameter is from 7.56 ± 0.29 μm to 9.82 ± 0.30 μm in queens, from 6.06 ± 0.65 μm to 7.91 ± 0.20 μm in workers, and from 6.12 ± 00.31 μm to 10.22 ± 0.48 μm in males (Table 4). 

#### 3.2.3. Basiconica Sensilla (BaS)

BaS are coniform, short, and stout, and the surface is smooth, with an orbicular basal fossa (Figure 4H). They are distributed on all sides from F4 to F10 of queens and workers (extending to F3 in *B. terrestris*) (Figure 6, Table 3). In the same species, the greatest number of BaS is in queens, more than workers (Table 3). In the same caste, the number of BaS *B. breviceps* is the most in queens, significantly more than other species; in workers, the number of BaS *B. terrestris* is the most, having no significant differences among the four species (*p >* 0.05, Table 3). The length of sensilla BaS is from 10.61 ± 0.79 μm to 11.74 ± 0.90 μm in queens and 9.82 ± 0.35 μm to 14.95 ± 0.25 μm in workers (Table 4). The width of basal diameter is from 3.63 ± 0.16 μm to 5.44 ± 0.19 μm in queens and from 3.69 ± 0.38 μm to 4.05 ± 0.20 μm in workers (Table 4).

#### 3.2.4. Coeloconica Sensilla (COS)

COS are hole-like structures on the cuticle’s surface, with floccules in the hole from the front view, and two types, COS A and COS B, were observed according to their morphological features (Figure 5A–C). COS A has a big aperture 0.64–1.50 μm, and the edge of the hole convex (Figure 5B), while COS B has a small aperture, 0.62–0.94 μm, with no bulge at the hole edge (Figure 5C). Two types of sensilla are both distributed in all or partial flagellomeres and mainly distributed in the inner, outside, and dorsal side of F3–F10, less in the ventral side of the terminal segment, and both interactive emergence (Figure 6, Table 3). In the same species, the greatest number of COS A is mainly in the queen of the three species, while in workers of *B. flavescens* (Table 3) the greatest number of COS B is similar to COS A; however, it is different in males of *B. atripes*. In the same caste, for COS A, the number of *B. breviceps* is significantly more than other species in queens (*F* = 47.516, df = 3, *p* < 0.0001) and males (*F* = 8.452, df = 3, *p* < 0.001); in workers, the number of COS A in *B. flavescens* is the most, significantly more than the other species (*F* = 7.982, df = 3, *p* < 0.002); in males, the number of COS A in *B. flavescens* is the least, significantly less than the other species (*F* = 8.452, df = 3, *p* = 0.01, Table 3). For COS B, the number trend is mostly similar to COS A. For COS A, the width of basal diameter is from 0.98 ± 0.07 μm to 1.50 ± 0.08 μm in queens, from 0.64 ± 0.04 μm to 1.10 ± 0.09 μm in workers, and from 0.83 ± 0.12 μm to 1.04 ± 0.04 μm in males; for COS B, the width of basal diameter is from 0.64 ± 0.03 μm to 0.94 ± 0.06 μm in queens, from 0.62 ± 0.04 μm to 0.76 ± 0.02 μm in workers, and from 0.64 ± 0.04 μm to 0.90 ± 0.03 μm in males (Table 4).

#### 3.2.5. Chaetic Sensilla (CS)

CS are spine-like in shallow fossa, short and thin, straight, with distinct longitudinal ridges on the surface, inclining to the antennal surface, and forming an angle of 20–30° (Figure 5D,E). According to their morphological features, there are two types of chaetic sensilla.

CS A has no extended structure at the base (Figure 5D). It is only distributed dorsal side in SC, PE, and F1 in three castes of all species (Figure 6, Table 3). In the same species, the greatest number of CS A is in workers, more than queens and males (except in *B. atripes*) (Table 3). In the same caste, the number of CS A in *B. atripes* is significantly more than other species in queens (*F* = 90.958, df = 3, *p* < 0.05, Table 3); in workers, the number of CS A in *B. atripes* is the most, significantly more than *B. breviceps* and *B. flavescens*; in males, the number of CS A in *B. terrestris* is the most, significantly more than *B. atripes* and *B. breviceps*. The length of sensilla CS A is from 35.73 ± 1.98 μm to 58.46 ± 1.64 μm in queens, from 43.03 ± 2.16 μm to 71.37 ± 7.37 μm in workers, and from 21.45 ± 1.06 μm to 30.01 ± 1.94 μm in males (Table 4). The width of basal diameter is from 3.75 ± 0.17 μm to 6.60 ± 0.25 μm in queens, from 4.01 ± 0.17 μm to 4.34 ± 0.20 μm in workers, and from 1.51 ± 0.06 μm to 2.88 ± 0.09 μm in males (Table 4).

CS B has an extended structure in basal 1/2 of the sensilla, connecting the body with the surface of the antennae, namely the extended basal structure of CS B (CS Bb) (Figure 5E,F). This type was first recorded and only distributed in dorsal side of F1 in the female of *B. atripes* (Figure 6, Table 3). The number of CS B in the queen is significantly more than in the workers (*t* = 5.014, df = 8, *p* < 0.05, Table 3). The length of CS B in *B. atripes* is 62.12 ± 1.63 μm in the queen, significantly more than workers (25.80 ± 0.73 μm) (*t* = 20.476, df = 12, *p* < 0.05, Table 4). The width of basal diameter is 6.46 ± 0.27 μm in the queen, significantly more than the workers (2.30 ± 0.08 μm) (*t* = 16.522, df = 14, *p* < 0.05, Table 4). The length of CS Bb is 13.37 ± 0.41 μm in queens, and there is no significant difference from workers (12.23 ± 0.48 μm) (*p >* 0.05; Table 4).

#### 3.2.6. Böhm Sensilla (BS)

BS are like trichodea sensilla TS C/D, mostly vertically oriented to the surface, without basal fossa at the base; however, they are shorter than TS C/D with a nearly smooth surface (Figure 5G,H). This type of BS is distributed on the inner and outer sides of PE among three castes of all species (Figure 6). In the same species, the greatest number of BS is mainly in males, while in the queen of *B. atripes* and in workers of *B. terrestris* (Figure 6, Table 3). In the same caste, the four species have no significant differences in queens and workers; in males, the number of BS in *B. flavescens* is the most, significantly more than *B. atripes* and *B. terrestris* (*p* < 0.05; Table 3). The length of sensilla BS is from 16.50 ± 1.40 μm to 19.62 ± 1.86 μm in queens, from 14.87 ± 1.17 μm to 21.23 ± 1.18 μm in workers, and from 19.74 ± 1.43 μm to 22.21 ± 2.62 μm in males (Table 4). The width of basal diameter is from 3.69 ± 0.14 μm to 4.60 ± 0.28 μm in queens, from 2.44 ± 0.12 μm to 3.47 ± 0.13 μm in workers, and from 2.85 ± 0.27 μm to 3.99 ± 0.13 μm in males (Table 4).

#### 3.2.7. Branched Setae (BrS)

BrS are long, branched hairs with longitudinal ridges, and born in the shallow fossa on the scape surface (Figure 5I–K). Two types of branched setae were observed on the inner and outer sides.

BrS A is short, with branched hairs extending in one direction, distributed in SC among three castes of all species (Figure 5I and Figure 6). In the same species, the greatest number of BrS A is in the workers of three species, while in the males of *B. atripes* (Table 3). In the same caste, the number of BrS A in *B. terrestris* is the least, significantly less than *B. atripes* and *B. breviceps* in queens; in workers, the number of BrS A in *B. atripes* is the least, significantly less than other species (*F* = 13.353, df = 3, *p* < 0.0001); in males, the number of BrS A in *B. flavescens* is the least, significantly less than *B. breviceps* and *B. terrestris* (*p* < 0.05). The length of sensilla BrS A is from 55.22 ± 4.56 μm to 166.80 ± 15.78 μm in queens, from 58.58 ±3.92 μm to 244.4 ±18.91 μm in workers, and from 48.30 ± 4.44 μm to 115.67 ± 5.21 μm in males (Table 4). The width of basal diameter is from 3.87 ± 0.13 μm to 11.51 ± 0.71 μm in queens, from 4.04 ± 0.21 μm to 6.23 ± 0.33 μm in workers, and from 4.41 ± 0.15 μm to 6.54 ± 0.31 μm in males. 

BrS B is long, with branched hairs extending in different directions, and is distributed in SC among three castes of three species (except in SC of *B. atripes* without BrS B) (Figure 5J,K and Figure 6). In the same species, the least number of BrS B is in queens among the three species. In the same caste, the number of BrS B in *B. flavescens* is the most in queens, but there is no significant difference among the three species (*p* > 0.05); in workers, the number of BrS B in *B. flavescens* is the least, significantly less than *B. breviceps* (*F* = 17.729, df = 3, *p* < 0.05); in males, the number of BrS B in *B. flavescens* is the most, significantly less than the other two species (*p* < 0.05). The length of sensilla BrS B is from 186.63 ± 20.86 μm to 326.00 ± 27.90 μm in queens, from 58.58 ± 3.92 μm to 244.40 ± 18.91 μm in workers, and from 243.00 ± 22.70 μm to 526.86 ± 33.35 μm in males. The width of basal diameter is from 5.86 ± 0.45 μm to 8.59 ± 0.50 μm in queens, from 4.04 ± 0.21 μm to 6.23 ± 0.33 μm in workers, and from 4.59 ± 0.16 μm to 7.79 ± 0.76 μm in males.

## 4. Discussion

This study compares antennal structures in three castes among four species of *Bombus* (Hymenoptera: Apidae), mainly including antennal length and sensilla. The results revealed the differences in antennal length, morphology, distribution, and number of sensilla, which may help taxon and identification of bumblebees, and provide the basis for the communication between inter- and intraspecific recognition, and coevolution between the bumblebees.

### 4.1. Antennal Length

For the antennal length of Hymenopteran, non-parasitic species, including bumblebees, differ significantly from parasitic species in that females have longer scape and shorter flagellum than males in 114 species of seven families [13]. Similarly, our results showed that the scape length in queens and workers is longer than in males; moreover, there are always significant differences in queens from males, and not always significant differences from workers. The length of the flagellum in the female is not always shorter than in the male, for example, the length in the queen of *B. flavescens* is significantly longer than in males. For pedicel and flagellomeres, the length varies among species and castes. The total length of the antenna in workers is less than the queen and male, and especially significantly different from the queen, which is relevant to small body size and fewer flagellomeres [13]. In addition, the length of the total antenna, flagellum, pedicel, and all flagellomeres in the queen of *B. flavescens* is significantly longer than that in males. It may be that the queen of *B. flavescens* has a larger body under the same conditions, and the body size proportional to antennae length. So, for antennal length, there are some taxonomic effects in identifying species and caste among bumblebees.

In our observation, the antenna length of males is longer than that of workers as a whole. An ecological basis for these differences in antenna dimensions may relate to sexual differences in searching behavior [13]. Longer antennae mean that male antennae have a larger surface area to accommodate more receptors to enhance the positioning of mates, which may be important because males usually compete fiercely to obtain sexually receptive females [40].

In this experiment, the total lengths of antennae and flagellum of different kinds of worker bees are different. Previous studies have shown that female non-parasitic bees use visual and olfactory cues to find flowers and collect food for their offspring and themselves [41,42]. The length of the antennae means the difference in the number of sensilla, especially olfactory receptors, such as placodea sensilla. Therefore, the length differences may indicate that the four species of workers have unique preferences in flower-visiting selection and taste perception.

### 4.2. Inter- and Intraspecific Differences in Antennal Sensilla 

Schneider indicated that insect antennae mainly have ten types of sensilla: sensilla scolopalia, sensilla campaniformia, sensilla squamiformia, sensilla styloconica, sensilla placodea, sensilla ampullaceal, sensilla coeloconica, sensilla basiconica, sensilla trichodea, and sensilla chaetica [15]. With the continuous development of scanning technology, more than 15 types of sensilla have been successively recorded in the antennae of different bumblebees [8,16,26,27]. In this study,13 subtypes belonging to six types of antennal sensilla were described among three castes of four species, *B. atripes*, *B. breviceps*, *B. flavescens*, and *B. terrestris*, including trichodea sensilla (TS A–E), placodea sensilla (PS A, B), basiconica sensilla (BaS), coeloconica sensilla (COS A, B), chaetic sensilla (CS A, B), and Böhm sensilla (BS). 

Trichodea sensilla (TS), as the most widely distributed and numerous sensilla in the antennae of insects [43], have been reported in Hymenoptera [44,45]. In this study, we found five subtypes of trichodea sensilla (TS A–E), which may have different functions. Previous research showed that sensilla trichodea A are typically thin- and single-walled hairs with wall pores in Colletidae by Transmission Electron Microscope (TEM), but in our study, wall pores were not observed by SEM [46]. For the distribution, TS A of *Bombus pauloensis* is distributed in F2 to F10 in workers, and F2 to F11 in males [8]. However, we observed that TS A is mainly distributed in each segment of the flagellum, and few of them are also distributed in PE, which may be related to the special role of this sensilla. Lacher found that the TS A in *Apis mellifera* did not respond to odor and other forms of stimulation [47], but then Esslen and Kaissling indicated that TS A has the function of smell perception [45]. For bumblebees, it is not known whether TS A is related to smell function, which needs to be further determined. Sensilla trichodea B are mechanoreceptors and sensilla trichodea C/D combines gustatory and tactile functions in 12 species of male bumblebees [16]. Sensilla E, also reported in *Bombus hypocrite* [27], is similar to trichodea sensilla B, the difference is that the apical part is curved and distributed in the end segment in this study. However, its function was not discussed. We speculate that TS E plays a specific role in contact between intraspecific and interspecific recognition due to its distribution on the terminal segment in the four species. 

Placodea sensilla (PS) are oval, with a constant line extending to the center of the disc, which has been reported in Hymenoptera [48,49]. In this study, two types of placodea sensilla were found, have a difference in PS A with a narrow border of the shallower disc, and PS B with a wide border of the deeper disc; differences in morphology were not caused by human factors because both are relatively staggered and adjacent. However, PS A is more widely distributed than PS B, and the number of PS A is far more than PS B. Previous studies have found placodea sensilla in 12 species of European male bumblebees [16], *Bombus pauloensis* [8] and *Apis mellifera* [48], which are similar to our PS A. PS A of *Bombus pauloensis* is distributed F2 to F9 in the workers, and F2 to F11 in males, and there is a difference in distribution pattern in the four species, which may lead to different sensory abilities among species. In Hymenoptera, PS A has remote chemical positioning functions [50,51] and is also sensitive to the pheromone of the queen and the odor of Nasanov gland by providing channels for external volatile substances to enter the antenna [52]. In addition, placodea sensilla A in males of Hymenoptera may be relevant to the perception of females and plant odors [53], which is also reflected in *Apis mellifera*, with a higher amount placodea sensilla A in males than queens and workers [54]. In general, we observed that the number of placodea sensilla A in males is more than that of females, which may be related to the above functions. We speculate that PSA may have a function related to olfaction, and have sex dimorphism. The function difference between PSA and PSB is not clear now, whether PS B and PS A have the same function needs further study. 

Previous studies have proposed that the BaS is only distributed in females [16,27,43,45] and has different subtypes [27,55]. We found only one type of BaS in females, and none in males. In *Bombus hypocrite*, it is distributed in F3–F10 of workers [27]. In *Bombus pauloensis*, BaS are distributed in F8 to F10 of the queen, and F3 to F10 of the workers [8]. In our study, the sensilla are distributed in F3 to F10 on female antennae of *B. terrestris*, and the other three females are all distributed in F4 to F10. The sensilla have abundant small holes and nerve cells inside, so it can recognize smell [56], it may be a kind of olfactory sensilla which can sense plant odor stimulation [53,57], which is helpful for insects to find and locate nectariferous plants [58]. Based on the characteristics of the BaS we found, we speculate that basiconica sensilla play a similar role in the four bumblebee species. However, the difference in the specific functions, such as flower-visiting preference in the four species, needs further study.

Coeloconica sensilla (COS) are pore-like structures, which have two types, COS A with a large aperture and the edge of the hole convex, and COS B with a small aperture and no bulge at the hole edge. COS A has been reported in honeybees and bumblebees [16,59], with a wide cavity with a central short peg and encircled by folds [16]. In *Bombus pauloensis*, COS A is distributed from F3 to the end segment in females, and from F7 to F11 in drones [8]; in *Camponotus japonicus*, COS A is distributed from F2 to F9 in workers and queens, while it is distributed from F11 in drones [60]; whereas in the honeybee, COS A spaces are not distributed in F1 and F2 [45,61]; in *Bombus hypocrite*, these sensilla are distributed in the scape, pedicel, and flagellum, while we observed that the sensilla are only distributed in the flagellum, and the distribution characteristics are generally similar to those of *B. hypocrite* and *B. pauloensi*, but there are some differences between species such as the number of the COS A in the queen is the most and the workers is the least in most species, while the number of COS A in workers in *B. flavescens* is the most. Some researchers deem COS A is a thermo- and hygroreceptor [16,62]. It is a small aperture similar to ampullaceal sensilla according to previous research [16,27], and it has a long tube at the bottom, but we did not observe it. The function of COS B is not clear, and it may be a thermoreceptor responding to high and low humidity [63], or CO_2_-sensitive [47]. This may indicate that the four bumblebees in this study have differences in their perception of temperature, humidity, and carbon dioxide. 

In this study, chaetic sensillum (CS) has two subtypes, CS A and CS B, in four bumblebee species, of which CS B was first found. The previous study showed that there were about four subtypes of CS recorded in Hymenoptera, Coleoptera and Lepidoptera [64,65,66], of which CS A is thorn-like and as a common subtype is consistent with the chaetic sensilla in *B. hypocrite* [27], sensilla chaetica type 4 in *Epidinocarsis lopezi* and *Leptomastix dactylopii* [67], Ch.1 in *Xylotrechus quadripes* [68], SC.1 in *Aromia bungii* [65], and SC.S in *Mythimna separata* [66]. The function of CS A sensing mechanical stimuli in *B. hypocrite* [27] and antennal contact in *Epidinocarsis lopezi* and *Leptomastix dactylopii* (Hymenoptera: Encyrtidae) have been recorded [67]. Additionally, CS A can protect the olfactory sensilla in *Xylotrechus quadripes* and support protection in *Aromia bungii* (Coleoptera: Cerambycidae) [65]. In Lepidoptera, this sensillum is a mechanoreceptor in *Mythimna separata* of the family Noctuidae [66]. Therefore, we speculate that CS A has a mechanoreceptor function. CS B is similar to CS A, but with an extended skirt band at the base and only distributed in *B. atripes* females. The operation mechanism of CS B is unclear but may be related to some mechanical functions of females. 

Böhm sensilla (BS) have been reported in Thripidae [69], Pyralidae [70], Cerambycidae [68], Lepidoptera, and most insects [71] and also observed in *Bombus* [26], which has the same distribution position in the junction of scape and pedicel. BS function as proprioceptors which monitor the antennal movements and position [71,72]. Behavioral studies have shown that removing BS leads to continuous antenna collision, which means these sensilla participate in mediating antenna entry into flight positioning [73]. Based on our observation results, we speculate that the sensor may participate in localization.

Branched setae (BrS) are bud-like, with small branches on the surface in one or different directions. Branched setae A (BrS A) and branched setae B (BrS B) in this study are like two subtypes of the bud-like sensilla, Sbl C and Sbl A of *B. hypocrita*, respectively [27]. However, some authors think branched setae are feathery bristles without nerves [8,15,16]. So, in this paper, branched setae (BrS) are not treated as sensilla.

Overall, we did not find sensilla campaniformia, which occurs in Apidae, even in bumblebees [13,16,27,48]. In contrast, chaetic sensillum B (CS B), a new type, was first recorded in Apidae, but the specific functions of sensilla are not clear.

## 5. Conclusions

This study provided the antennal structures and morphology of three castes of the four *Bombus* species (*B. atripes*, *B. breviceps*, *B. flavescens*, and *B. terrestris*), with focus on the length of antennal segments and the types, distribution, and number of antennal sensilla. The result shows that the length of each segment and total length are used to identify species and caste and the coevolution relationship between antennae, and 13 types of antennal sensilla in total were identified among four species, including five types of trichodea sensilla (TS A–E), two types of placodea sensilla (PS A, PS B), basiconica sensilla (BaS), and coeloconica sensilla (COS A, COS B), two types of chaetic sensilla (CS A, CS B), and Böhm sensilla (BS), and two types of branched setae (BrS A, BrS B), of which chaetic sensilla B (CS B) was firstly recorded in bumblebees.

## Figures and Tables

**Table 1:** The queens’ sources of four species of *Bombus*.

Species	Source	Location
*B. terrestris*	Bought from Zhongnong Fengshou Ecological Agricultural Technology (Tianjin, China) Co., Ltd.	Tianjin City, China
*B. breviceps*	Collected in the field	Zunyi City, China (28.323° N, 105.481° E)
*B. atripes*	Collected in the field	Guiyang City, China (26.509° N, 106.534° E)
*B. flavescens*	Collected in the field	Guiyang City, China (26.509° N, 106.534° E)

**Table 2:** The mean length of each flagellomere in the same caste and species of four Bumblebee species (μm ± SE).

Castes	Segments	*B. atripes*	*B. breviceps*	*B. flavescens*	*B. terrestris*
Queen	F1	594.20 ± 17.85 ^a^	659.30 ± 5.55 ^a^	609.20 ± 18.98 ^ab^	605.40 ± 16.94 ^a^
	F2	380.00 ± 4.81 ^d^	386.50 ± 7.81 ^c^	542.00 ± 3.58 ^b^	415.20 ± 4.73 ^c^
	F3	478.00 ± 10.93 ^bc^	456.30 ± 4.07 ^bc^	646.70 ± 10.40 ^a^	464.00 ± 5.78 ^b^
	F4	484.20 ± 4.02 ^bc^	450.80 ± 8.52 ^bc^	659.60 ± 10.10 ^a^	462.80 ± 10.41 ^b^
	F5	473.20 ± 3.65 ^bc^	460.30 ± 5.56 ^b^	647.30 ± 7.46 ^a^	428.40 ± 5.94 ^bc^
	F6	467.10 ± 5.69 ^c^	465.10 ± 9.47 ^b^	646.80 ± 12.54 ^a^	415.80 ± 4.30 ^c^
	F7	470.40 ± 5.71 ^bc^	436.80 ± 12.71 ^bc^	630.90 ± 15.49 ^a^	429.80 ± 4.92 ^bc^
	F8	452.20 ± 5.92 ^c^	435.20 ± 13.25 ^bc^	616.30 ± 12.83 ^a^	415.40 ± 6.56 ^c^
	F9	453.30 ± 8.32 ^c^	414.40 ± 12.79 ^bc^	625.60 ± 19.05 ^a^	427.00 ± 7.38 ^bc^
	F10	513.40 ± 9.49 ^b^	435.10 ± 32.76 ^bc^	617.70 ± 19.06 ^a^	442.40 ± 6.70 ^bc^
Worker	F1	522.50 ± 4.65 ^a^	554.90 ± 6.59 ^a^	659.10 ± 6.59 ^a^	531.20 ± 6.34 ^a^
	F2	262.00 ± 6.62 ^e^	350.80 ± 8.87 ^d^	435.30 ± 5.43 ^ab^	330.10 ± 11.32 ^c^
	F3	347.30 ± 8.36 ^cd^	421.20 ± 6.24 ^bc^	490.30 ± 5.57 ^a^	471.00 ± 8.19 ^b^
	F4	361.30 ± 3.92 ^cd^	402.20 ± 8.52 ^c^	494.50 ± 6.50 ^a^	479.70 ± 10.05 ^b^
	F5	382.00 ± 13.44 ^c^	392.30 ± 6.83 ^cd^	474.60 ± 10.54 ^a^	450.80 ± 4.93 ^b^
	F6	391.80 ± 3.29 ^c^	397.60 ± 4.04 ^c^	476.00 ± 14.06 ^a^	444.10 ± 5.28 ^b^
	F7	364.80 ± 17.49 ^c^	379.70 ± 5.55 ^cd^	442.10 ± 13.67 ^ab^	484.10 ± 5.26 ^b^
	F8	351.90 ± 17.16 ^cd^	380.80 ± 4.23 ^cd^	444.20 ± 11.31 ^ab^	455.70 ± 8.78 ^b^
	F9	310.80 ± 6.52 ^de^	353.10 ± 3.50 ^d^	402.80 ± 16.6 ^b^	462.40 ± 8.65 ^b^
	F10	451.90 ± 5.08 ^b^	459.00 ± 16.20 ^b^	491.50 ± 17.59 ^a^	441.70 ± 10.57 ^b^
Male	F1	334.40 ± 7.69 ^f^	383.90 ± 7.19 ^d^	694.60 ± 4.75 ^a^	520.40 ± 7.52 ^a^
	F2	454.50 ± 5.77 ^bcd^	307.40 ± 4.51 ^e^	472.90 ± 5.38 ^e^	333.10 ± 9.25 ^e^
	F3	470.70 ± 6.69 ^abc^	535.20 ± 8.94 ^abc^	525.00 ± 6.10 ^cd^	436.60 ± 5.27 ^cd^
	F4	487.40 ± 7.69 ^ab^	496.60 ± 7.09 ^c^	529.50 ± 8.41 ^cd^	448.50 ± 7.02 ^cd^
	F5	479.60 ± 4.85 ^ab^	477.10 ± 4.67 ^c^	505.60 ± 11.77 ^cde^	468.30 ± 8.55 ^bc^
	F6	475.50 ± 4.89 ^ab^	526.60 ± 8.36 ^abc^	488.30 ± 12.21 ^cde^	438.00 ± 11.71 ^cd^
	F7	437.30 ± 4.67 ^cde^	505.90 ± 7.50 ^bc^	483.30 ± 9.02 ^de^	433.80 ± 9.26 ^cd^
	F8	433.60 ± 5.82 ^de^	524.60 ± 10.99 ^abc^	472.40 ± 12.02 ^e^	437.20 ± 5.68 ^cd^
	F9	410.80 ± 6.45 ^e^	507.30 ± 10.99 ^abc^	416.70 ± 3.12 ^f^	443.00 ± 4.79 ^cd^
	F10	425.00 ± 8.58 ^de^	550.70 ± 9.55 ^ab^	530.50 ± 4.50 ^c^	408.20 ± 6.67 ^d^
	F11	496.80 ± 4.20 ^a^	554.50 ± 13.13 ^a^	608.80 ± 11.65 ^b^	508.50 ± 9.02 ^ab^

Notes. Mean with the same letter is not significantly different (*p >* 0.05).

**Table 3:** Distribution and number of each type of antenna sensilla and branched setae in four *Bombus* species.

Castes and Types		Distribution				Number (Mean ± SE) (μm)			
		*B. atripes*	*B. breviceps*	*B. flavescens*	*B. terrestris*	*B. atripes*	*B. breviceps*	*B. flavescens*	*B. terrestris*
Q	TS A	F1, F2	F1, F2	F1, F2	F1, F2	70.80 ± 1.73 ^a^	57.00 ± 1.60 ^b^	79.80 ± 3.00 ^a^	43.20 ± 2.18 ^c^
	TS B	F2–F10	F2–F10	F5–F9	F3–F10	42.00 ± 3.69 ^a^	30.20 ± 1.11 ^b^	52.20 ± 1.58 ^a^	30.60 ± 1.93 ^b^
	TS C/D	F2–F10	PE, F1–F10	F1–F10	F3–F10	51.20 ± 1.95 ^b^	41.40 ± 2.66 ^c^	64.20 ± 2.14 ^a^	29.80 ± 1.31 ^d^
	TS E	F10	F10	F10	F10	28.60 ± 1.28 ^b^	31.60 ± 1.80 ^b^	44.60 ± 2.57 ^a^	15.40 ± 1.00 ^c^
	PS A	F1–F10	F2–F10	F2–F10	F2–F10	42.20 ± 1.11 ^d^	54.00 ± 1.17 ^c^	86.00 ± 2.12 ^b^	196.60 ± 4.80 ^a^
	PS B	F3–F8, F10	F3, F6, F7, F9	F2–F10	F2–F10	24.60 ± 0.83 ^c^	38.40 ± 1.37 ^d^	61.40 ± 1.82 ^b^	102.00 ± 6.47 ^a^
	BaS	F4–F10	F4–F10	F4–F10	F3–F10	6.00 ± 0.89 ^b^	14.80 ± 1.21 ^a^	6.60 ± 0.67 ^b^	9.00 ± 1.70 ^b^
	COS A	F2–F10	F1–F10	F3, F9	F3–F10	6.40 ± 0.73 ^b^	13.40 ± 0.61 ^a^	3.00 ± 0.57 ^c^	5.40 ± 0.36 ^bc^
	COS B	F2–F10	F1–F10	F3, F9	F3–F10	5.20 ± 0.77 ^b^	13.20 ± 2.46 ^a^	2.00 ± 0.49 ^b^	5.00 ± 0.94 ^b^
	CS A	SC, PE, F1	SC, PE, F1	SC, PE, F1	SC, PE, F1	88.40 ± 1.76 ^a^	36.40 ± 1.61 ^b^	23.20 ± 3.96 ^c^	44.60 ± 2.59 ^b^
	CS B	F1	-	-	-	65.80 ± 1.73 ^a^	-	-	-
	BS	PE	PE	PE	PE	12.80 ± 0.72 ^a^	15.80 ± 1.21 ^a^	14.00 ± 1.41 ^a^	11.00 ± 1.13 ^a^
	BrS A	SC	SC	SC	SC	17.80 ± 0.95 ^a^	14.80 ± 1.21 ^a^	13.00 ± 1.41 ^ab^	9.00 ± 0.85 ^b^
	BrS B	-	SC	SC	SC	-	4.00 ± 0.89 ^a^	6.80 ± 0.59 ^a^	6.00 ± 0.85 ^a^
W	TS A	F1, F2	F1, F2	F1, F2	F1, F2	61.80 ± 2.86 ^b^	109.00 ± 4.45 ^a^	74.80 ± 3.00 ^b^	56.00 ± 4.09 ^c^
	TS B	F2–F10	F2–F10	F5–F9	F3–F10	40.40 ± 0.96 ^c^	79.60 ± 2.05 ^a^	50.40 ± 2.05 ^b^	28.60 ± 1.19 ^d^
	TS C/D	F2–F10	PE, F1–F10	F1–F10	F3–F10	48.20 ± 1.11 ^b^	83.40 ± 3.28 ^a^	59.20 ± 2.11 ^b^	30.40 ± 3.95 ^c^
	TS E	F10	F10	F10	F10	27.60 ± 1.54 ^bc^	35.40 ± 2.13 ^ab^	40.60 ± 2.84 ^a^	20.20 ± 1.58 ^c^
	PS A	F1–F10	F2–F10	F2–F10	F2–F10	39.40 ± 1.87 ^c^	67.80 ± 1.56 ^b^	26.40 ± 0.78 ^d^	81.80 ± 3.00 ^a^
	PS B	F3–F8, F10	F3, F6, F7, F9	F2–F10	F2–F10	6.00 ± 0.63 ^c^	12.00 ± 1.72 ^c^	30.80 ± 1.99 ^a^	23.20 ± 1.11 ^b^
	BaS	F4–F10	F4–F10	F4–F10	F3–F10	5.00 ± 0.63 ^a^	6.00 ± 0.40 ^a^	4.80 ± 0.33 ^a^	6.80 ± 0.33 ^a^
	COS A	F2–F10	F1–F10	F3, F9	F3–F10	3.00 ± 0.28 ^cd^	2.20 ± 0.33 ^d^	5.00 ± 0.57 ^a^	4.00 ± 0.28 ^bc^
	COS B	F2–F10	F1–F10	F3, F9	F3–F10	3.80 ± 0.33 ^ab^	1.80 ± 0.33 ^b^	6.00 ± 1.06 ^a^	4.60 ± 0.36 ^a^
	CS A	SC, PE, F1	SC, PE, F1	SC, PE, F1	SC, PE, F1	62.80 ± 2.86 ^a^	46.20 ± 4.07 ^b^	47.40 ± 2.05 ^b^	53.40 ± 2.68 ^ab^
	CS B	F1	-	-	-	51.20 ± 1.95 ^b^	-	-	-
	BS	PE	PE	PE	PE	12.00 ± 1.72 ^a^	12.80 ± 1.21 ^a^	16.80 ± 1.43 ^a^	16.40 ± 1.25 ^a^
	BrS A	SC	SC	SC	SC	20.80 ± 0.72 ^b^	47.20 ± 1.48 ^a^	36.20 ± 5.09 ^a^	41.80 ± 1.56 ^a^
	BrS B	-	SC	SC	SC	-	24.60 ± 3.17 ^a^	14.80 ± 1.15 ^b^	18.60 ± 0.67 ^ab^
M	TS A	F1, F2	F1, F2	F1–F2	F1, F2	71.60 ± 2.48 ^b^	116.80 ± 3.36 ^a^	109.40 ± 7.01 ^a^	60.40 ± 2.01 ^b^
	TS B	F2–F10	F1–F10	F2–F10	F2–F10	46.20 ± 3.25 ^b^	79.60 ± 3.97 ^a^	68.80 ± 3.68 ^a^	29.60 ± 3.09 ^c^
	TS C/D	PE, F1–F10	F2–F10	F2–F10	F7–F10	51.20 ± 1.28 ^b^	84.40 ± 3.28 ^a^	83.20 ± 4.93 ^a^	43.80 ± 1.21 ^b^
	TS E	F11	F11	F11	F11	32.40 ± 1.37 ^b^	38.40 ± 1.61 ^bc^	79.60 ± 3.78 ^a^	25.20 ± 0.91 ^c^
	PS A	F2–F11	F4–F11	F2–F11	F4–F11	83.40 ± 4.04 ^b^	87.40 ± 1.87 ^b^	74.00 ± 2.67 ^b^	150.80 ± 10.43 ^a^
	PS B	F2–F11	F4–F11	F2–F11	F4–F11	30.60 ± 1.54 ^b^	12.80 ± 0.72 ^c^	11.80 ± 0.33 ^c^	45.00 ± 4.87 ^a^
	BaS	-	-	-	-	-	-	-	-
	COS A	F8–F11	F3, F4, F11	F9–F11	F6–F11	4.60 ± 0.83 ^b^	8.00 ± 0.85 ^a^	2.60 ± 0.46 ^b^	4.80 ± 0.52 ^b^
	COS B	F8–F11	F3, F4, F11	F9–F11	F6–F11	6.60 ± 1.28 ^a^	7.80 ± 0.72 ^a^	1.80 ± 0.33 ^b^	4.80 ± 1.25 ^ab^
	CS A	SC, PE, F1	SC, PE, F1	SC, PE, F1	SC, PE, F1	28.60 ± 1.80 ^b^	27.60 ± 1.28 ^b^	32.00 ± 1.44 ^ab^	35.20 ± 0.77 ^a^
	CS B	-	-	-	-	-	-	-	-
	BS	PE	PE	PE	PE	11.00 ± 1.44 ^b^	17.80 ± 1.75 ^ab^	20.60 ± 2.01 ^a^	12.20 ± 1.11 ^b^
	BrS A	SC	SC	SC	SC	28.60 ± 1.28 ^bc^	38.40 ± 2.39 ^a^	21.00 ± 2.26 ^c^	31.20 ± 0.72 ^ab^
	BrS B	-	SC	SC	SC	-	12.80 ± 0.95 ^b^	31.60 ± 3.62 ^a^	15.80 ± 0.59 ^b^

Notes. Q: queen; W: worker; M: male. Mean with the same letter are not significantly different (*p >* 0.05). Significant differences in sensilla number between different species are indicated by horizontal a–d, while CS B of *B. atripes* is indicated by vertical a and b.

**Table 4:** The length and basal diameter of antennae sensilla and branched setae in four *Bombus* species (Mean ± SE).

Castes and Types		Length (μm)				Basal Diameter (μm)			
		*B. atripes*	*B. breviceps*	*B. flavescens*	*B. terrestris*	*B. atripes*	*B. breviceps*	*B. flavescens*	*B. terrestris*
Q	TS A	22.47 ± 1.36 ^b^	31.57 ± 1.16 ^a^	26.9 ± 0.94 ^ab^	26.34 ± 2.18 ^ab^	4.84 ± 0.38 ^a^	4.53 ± 0.09 ^ab^	3.69 ± 0.19 ^bc^	3.24 ± 0.21 ^c^
	TS B	19.89 ± 0.81 ^a^	11.06 ± 1.04 ^c^	16.18 ± 0.86 ^b^	17.11 ± 0.41 ^ab^	3.12 ± 0.17 ^a^	2.84 ± 0.22 ^a^	1.41 ± 0.07 ^b^	2.63 ± 0.12 ^a^
	TS C/D	21.71 ± 1.36 ^b^	26.83 ± 1.30 ^b^	23.93 ± 0.88 ^b^	34.03 ± 2.09 ^a^	2.31 ± 0.15 ^b^	4.29 ± 0.23 ^a^	2.62 ± 0.21 ^b^	3.78 ± 0.18 ^a^
	TS E	-	-	-	-	1.84 ± 0.09 ^a^	1.74 ± 0.07 ^ab^	1.44 ± 0.09 ^b^	1.7 ± 0.09 ^ab^
	PS A	14.48 ± 0.39 ^a^	13.96 ± 0.24 ^a^	14.80 ± 0.34 ^a^	14.86 ± 0.24 ^a^	10.14 ± 0.40 ^a^	10.32 ± 0.29 ^a^	9.83 ± 0.23 ^a^	10.38 ± 0.35 ^a^
	PS B	12.02 ± 0.31 ^bc^	14.19 ± 0.48 ^a^	13.30 ± 0.29 ^ab^	11.36 ± 0.21 ^c^	7.56 ± 0.29 ^b^	9.82 ± 0.30 ^a^	8.94 ± 0.41 ^ab^	8.03 ± 0.29 ^b^
	BaS	11.74 ± 0.9 ^a^	10.61 ± 0.79 ^a^	11.21 ± 0.53 ^a^	11.35 ± 0.50 ^a^	3.77 ± 0.13 ^c^	4.57 ± 0.16 ^b^	3.63 ± 0.16 ^c^	5.44 ± 0.19 ^a^
	COS A	-	-	-	-	1.11 ± 0.02 ^ab^	1.20 ± 0.09 ^ab^	0.98 ± 0.07 ^b^	1.50 ± 0.08 ^a^
	COS B	-	-	-	-	0.64 ± 0.03 ^b^	0.86 ± 0.05 ^ab^	0.71 ± 0.04 ^ab^	0.94 ± 0.06 ^a^
	CS A	49.63 ± 2.59 ^a^	58.46 ± 1.64 ^a^	51.74 ± 3.68 ^a^	35.73 ± 1.98 ^b^	6.18 ± 0.35 ^a^	6.60 ± 0.25 ^a^	4.50 ± 0.32 ^b^	3.75 ± 0.17 ^b^
	CS B	62.12 ± 1.63 ^a^	-	-	-	6.46 ± 0.27 ^a^	-	-	-
	CS Bb	13.37 ± 0.41 ^a^	-	-	-	-	-	-	-
	BS	19.62 ± 1.86 ^a^	16.88 ± 1.04 ^a^	16.5 ± 1.4 ^a^	17.96 ± 0.84 ^a^	4.36 ± 0.31 ^a^	4.60 ± 0.28 ^a^	3.69 ± 0.14 ^a^	4.06 ± 0.23 ^a^
	BrS A	166.8 ± 15.78 ^a^	68.04 ± 3.37 ^b^	92.33 ± 7.27 ^ab^	55.22 ± 4.56 ^b^	11.51 ± 0.71 ^a^	6.36 ± 0.3 ^b^	3.87 ± 0.13 ^c^	5.74 ± 0.55 ^bc^
	BrS B	-	232.81 ± 13.42 ^b^	326.00 ± 27.9 ^a^	186.63 ± 20.86 ^b^	-	8.59 ± 0.5 ^a^	5.86 ± 0.45 ^b^	7.21 ± 0.53 ^ab^
W	TS A	17.24 ± 1.28 ^c^	23.31 ± 0.81 ^b^	29.87 ± 1.67 ^a^	17.53 ± 1.11 ^c^	3.17 ± 0.20 ^ab^	4.12 ± 0.28 ^a^	2.72 ± 0.19 ^b^	3.30 ± 0.17 ^ab^
	TS B	20.71 ± 0.95 ^b^	25.80 ± 1.87 ^a^	13.30 ± 0.37 ^c^	21.00 ± 0.83 ^b^	1.80 ± 0.09 ^ab^	2.17 ± 0.10 ^a^	1.45 ± 0.09 ^b^	1.85 ± 0.07 ^a^
	TS C/D	17.79 ± 0.73 ^b^	29.05 ± 0.58 ^a^	32.25 ± 1.47 ^a^	18.04 ± 0.63 ^b^	2.83 ± 0.22 ^ab^	3.47 ± 0.11 ^a^	2.26 ± 0.19 ^b^	2.90 ± 0.21 ^ab^
	TS E	-	-	-	-	1.51 ± 0.09 ^b^	1.93 ± 0.1 ^a^	1.29 ± 0.07 ^b^	1.58 ± 0.06 ^ab^
	PS A	12.74 ± 0.34 ^b^	13.37 ± 0.31 ^b^	15.82 ± 0.33 ^a^	12.99 ± 0.32 ^b^	8.38 ± 0.17 ^a^	9.42 ± 0.34 ^a^	9.06 ± 0.24 ^a^	8.52 ± 0.16 ^a^
	PS B	14.47 ± 0.18 ^a^	12.40 ± 0.40 ^b^	14.39 ± 0.18 ^a^	14.44 ± 0.20 ^a^	7.78 ± 0.20 ^a^	6.06 ± 0.65 ^b^	7.78 ± 0.20 ^a^	7.91 ± 0.20 ^a^
	BaS	14.70 ± 0.25 ^a^	9.82 ± 0.35 ^b^	9.89 ± 0.65 ^ab^	14.95 ± 0.25 ^a^	3.72 ± 0.13 ^a^	3.69 ± 0.38 ^a^	4.05 ± 0.20 ^a^	3.82 ± 0.11 ^a^
	COS A	-	-	-	-	0.64 ± 0.04 ^b^	1.09 ± 0.08 ^a^	1.10 ± 0.09 ^a^	0.65 ± 0.04 ^b^
	COS B	-	-	-	-	0.62 ± 0.04 ^a^	0.76 ± 0.02 ^a^	0.73 ± 0.03 ^a^	0.66 ± 0.04 ^a^
	CS A	43.03 ± 2.16 ^b^	62.50 ± 1.60 ^a^	71.37 ± 7.37 ^a^	43.90 ± 1.77 ^b^	4.17 ± 0.16 ^a^	4.34 ± 0.20 ^a^	4.01 ± 0.17 ^a^	4.34 ± 0.12 ^a^
	CS B	25.80 ± 0.73 ^b^	-	-	-	2.30 ± 0.08 ^b^	-	-	-
	CS Bb	12.23 ± 0.48 ^a^	-	-	-	-	-	-	-
	BS	14.87 ± 1.17 ^b^	18.32 ± 1.09 ^ab^	21.23 ± 1.18 ^a^	15.37 ± 0.96 ^b^	3.32 ± 0.23 ^a^	2.44 ± 0.12 ^b^	3.35 ± 0.23 ^a^	3.47 ± 0.13 ^a^
	BrS A	14.70 ± 0.25 ^a^	9.82 ± 0.35 ^b^	9.89 ± 0.65 ^ab^	14.95 ± 0.25 ^a^	3.72 ± 0.13 ^a^	3.69 ± 0.38 ^a^	4.05 ± 0.20 ^a^	3.82 ± 0.11 ^a^
	BrS B	-	67.99 ± 4.22 ^b^	58.58 ± 3.92 ^b^	244.40 ± 18.91 ^a^	-	5.23 ± 0.22 ^ab^	4.04 ± 0.21 ^b^	6.23 ± 0.33 ^a^
M	TS A	21.18 ± 0.98 ^a^	21.19 ± 0.93 ^a^	20.04 ± 1.02 ^a^	23.82 ± 1.31 ^a^	3.75 ± 0.31 ^ab^	3.8 ± 0.22 ^ab^	2.89 ± 0.08 ^b^	4.63 ± 0.21 ^a^
	TS B	13.7 ± 0.93 ^ab^	13.58 ± 0.75 ^ab^	11.04 ± 0.48 ^b^	15.46 ± 0.63 ^a^	1.71 ± 0.11 ^a^	1.79 ± 0.09 ^a^	1.89 ± 0.10 ^a^	1.96 ± 0.09 ^a^
	TS C/D	22.73 ± 0.86 ^a^	22.81 ± 0.76 ^a^	18.63 ± 0.33 ^b^	21.30 ± 1.54 ^ab^	2.48 ± 0.16 ^a^	2.64 ± 0.11 ^a^	2.21 ± 0.06 ^a^	2.85 ± 0.22 ^a^
	TS E	-	-	-	-	1.70 ± 0.18 ^a^	1.82 ± 0.07 ^a^	1.20 ± 0.08 ^b^	1.73 ± 0.08 ^a^
	PS A	13.37 ± 0.32 ^a^	13.40 ± 0.24 ^a^	12.77 ± 0.34 ^a^	13.09 ± 0.28 ^a^	7.88 ± 0.35 ^c^	8.25 ± 0.24 ^bc^	9.53 ± 0.34 ^ab^	9.61 ± 0.37 ^a^
	PS B	9.01 ± 0.60 ^b^	8.87 ± 0.49 ^b^	13.57 ± 0.57 ^a^	11.82 ± 0.41 ^a^	6.41 ± 0.36 ^bc^	6.12 ± 0.31 ^c^	10.22 ± 0.48 ^a^	7.70 ± 0.14 ^b^
	BaS	-	-	-	-	-	-	-	-
	COS A	-	-	-	-	0.83 ± 0.12 ^a^	0.85 ± 0.08 ^a^	0.99 ± 0.09 ^a^	1.04 ± 0.04 ^a^
	COS B	-	-	-	-	0.78 ± 0.07 ^a^	0.64 ± 0.04 ^a^	0.79 ± 0.08 ^a^	0.90 ± 0.03 ^a^
	CS A	21.45 ± 1.06 ^b^	21.94 ± 1.11 ^b^	28.21 ± 0.78 ^a^	30.01 ± 1.94 ^a^	2.69 ± 0.20 ^a^	2.74 ± 0.12 ^a^	1.51 ± 0.06 ^b^	2.88 ± 0.09 ^a^
	CS B	-	-	-	-	-	-	-	-
	CS Bb	-	-	-	-	-	-	-	-
	BS	21.07 ± 1.82 ^a^	21.59 ± 1.55 ^a^	22.21 ± 2.62 ^a^	19.74 ± 1.43 ^a^	2.85 ± 0.27 ^b^	2.91 ± 0.16 ^b^	3.34 ± 0.15 ^ab^	3.99 ± 0.13 ^a^
	BrS A	115.67 ± 5.21 ^a^	110.67 ± 5.62 ^a^	48.30 ± 4.44 ^c^	79.86 ± 5.71 ^b^	6.48 ± 0.32 ^a^	6.54 ± 0.31 ^a^	4.41 ± 0.15 ^b^	6.20 ± 0.38 ^a^
	BrS B	-	334.29 ± 31.81 ^b^	526.86 ± 33.35 ^a^	243.00 ± 22.70 ^b^	-	7.79 ± 0.76 ^a^	6.54 ± 0.44 ^a^	4.59 ± 0.16 ^b^

Notes. Q: queen; W: worker; M: male. Means with the same letter are not significantly different (*p >* 0.05). Significant difference indicates a horizontal comparison and in CS B and CS Bb between queens and workers of *B. atripes*. Length of TS E was not measured.

## Data Availability

Original data at https://doi.org/10.6084/m9.figshare.21849144.v1 (accessed on 23 February 2023).
